# Major Maternal Dietary Patterns during Early Pregnancy and Their Association with Neonatal Anthropometric Measurement

**DOI:** 10.1155/2018/4692193

**Published:** 2018-05-31

**Authors:** Hossein Hajianfar, Ahmad Esmaillzadeh, Awat Feizi, Zahra Shahshahan, Leila Azadbakht

**Affiliations:** ^1^Food Security Research Center, Isfahan University of Medical Sciences, Isfahan, Iran; ^2^Research Committee of Community Nutrition, School of Nutrition and Food Sciences, Isfahan University of Medical Sciences, Isfahan, Iran; ^3^Department of Nutrition, School of Nutrition and Food Sciences, Semnan University of Medical Sciences, Semnan, Iran; ^4^Department of Community Nutrition, School of Nutritional Sciences and Dietetics, Tehran University of Medical Sciences, Tehran, Iran; ^5^Departments of Biostatistics and Epidemiology, School of Health, Isfahan University of Medical Sciences, Isfahan, Iran; ^6^Department of Gynecology, School of Medicine, Isfahan University of Medical Sciences, Isfahan, Iran

## Abstract

**Background:**

Anthropometric measurements of newborn infant are widely assessed as determinants of maternal nutrition. Although earlier studies have mostly examined the effects of particular nutrients or foods during gestational period on neonatal anthropometric measurements, there are few studies regarding the association of dietary patterns and mentioned measurements. So, the purpose of the current study was to investigate the association between major maternal dietary patterns and neonatal anthropometric measurements including body weight, head circumference, and height.

**Methods:**

The current prospective observational study is based on the data collected from 812 pregnant women. Dietary data was collected using a validated semiquantitative food frequency questionnaire.

**Results:**

Three identified major dietary patterns according to the results obtained from the factor loading matrix were (i) “western dietary pattern”; (ii) “traditional dietary pattern”; (iii) “healthy dietary pattern”. Overall, this study demonstrated a positive significant association between high adherences to western dietary pattern and chance of having low birth weight infant. However, such associations were not seen in women taking healthy and traditional dietary patterns.

**Conclusion:**

We found that healthier maternal dietary patterns during early pregnancy might be associated with lower risk of low birth weight. Further studies are required to confirm these findings.

## 1. Introduction

Anthropometric measurements, including body weight, head circumference, and height of newborn infants, are widely assessed as determinants of impaired fetal growth, intrauterine environment, and maternal nutrition [1]. Restricted fetal growth is one of the most important global public health problems which provides a foundation for developing chronic diseases throughout their life [2]. Low birth weight infants are susceptible to higher risk of developing iron deficiency anemia leading to impaired development and altered longer term neurodevelopment [3]. Also, impaired fetal growth, in particular in head circumference, is associated with nonoptimal neurodevelopmental outcome [4]. There are also several evidences regarding the association between the impaired growth indices at birth and increased risk of developing some chronic disorders such as obesity, diabetes [5], cardiovascular diseases [5], endothelial dysfunction [6], nonalcoholic fatty liver disease [7], and chronic kidney disease [8].

In addition to genetic factors [9], placenta structural [10] and environmental factors affect fetal growth in utero [9], and maternal nutritional status could impact on fetal growth and development [11]. There is increasing evidence that nutrients [12] and some foods [13, 14] play an important role in the fetal growth. Earlier studies have also shown that high-quality diet in the first trimester of pregnancy is positively associated with birth size including birth weight and birth length [15].

However, due to a wide range of nutrient interactions, maternal dietary patterns should be explored to achieve the association between maternal nutrition and fetal growth. It is noteworthy that there are few studies assessing the birth anthropometric measurements along the maternal dietary patterns [16].

Dietary patterns are different based on cultural, geographical, and regional influence in each area and can have effects on health outcomes [17, 18]. Limited data are available regarding the association of maternal dietary patterns and neonatal anthropometric measurements in Middle Eastern countries, where mentioned factors are different from western population [19].

So, the purpose of the current study was to investigate the association between major maternal dietary patterns during the first-trimester period and neonatal anthropometric measurements including body weight, head circumference, and height. Such findings will be used to implement informative interventional programs to control impaired fetal growth and develop practical policies to improve the diet quality among pregnant women.

## 2. Methods

### 2.1. Study Design and Participants

The current prospective observational study was conducted among pregnant women during the first trimester, who were being attended at health centers across Isfahan city in the central part of Iran during 2015-2016. A random sample of 812 pregnant women was selected from 20 various health centers by the multistage cluster random sampling method. Eligible criteria included singleton pregnant women during the first trimester without any medical condition, use of medications, and without following a specific diet. Exclusion criteria were avoiding of follow-up during the study and current-smoker women. Also we exclude twin pregnancies.

Informed consent was obtained from all participants. This study was approved by the research council (research project number: 193053) and ethics committee (research ethics number: IR.MUI.REC193053).

### 2.2. Data Collection

#### 2.2.1. Assessment of Dietary Intake

Pregnant women in these analyses completed validated 117-item semiquantitative food frequency questionnaire (FFQ) in the first visit, at 8–16 weeks. The validity and reliability of FFQ had been previously evaluated [20]. We inquired about the consumption of each food item, based on commonly used units or portion sizes, over the preceding 12 months on a daily, weekly, or monthly basis. Participants were asked to report their dietary intakes of foods based on nine multiple choice frequency response categories varying from “never or less than once a month” to “12 or more times per day”. Portion sizes of consumed foods were converted to grams from household measures. Supplements were also included to assess total nutrient intake. Then nutrient and energy intakes were computed by using NUTRITIONIST IV software (version 7.0; N-Squared Computing, Salem, OR), which was designed for Iranian foods. All nutrient values were energy-adjusted using the residuals method.

#### 2.2.2. Determination of Dietary Patterns

We applied principal component analysis in order to find major dietary patterns. Food items similar in nutrient profile were combined into 33 predefined food groups (Table 1). When the item was unique in the nutrient profile, it was not combined (e.g., salt). The factors were rotated by varimax rotation function. Correlated variables are aggregated by factor analysis. The obtained factors are linear combinations of the included variables, explaining as much variation in the original variables as possible. Three factors were retained, based on the screen plot, an eigenvalue of more than 1.9, and the interpretability of the derived factors and of the earlier literature.

Based on our interpretation of the data and of the earlier literature, these 3 factors were labeled as the healthy, western, and traditional patterns. The individual scores for the 3 patterns show the values estimated for each individual based on their consumption of foods and the factor loadings of the foods.

#### 2.2.3. Assessment of Other Variables

For the infants, birth date, gestational age, and anthropometric measurements including body weight, head circumference, and height were recorded at birth. Neonatal anthropometric measurements were categorized according to WHO standards and as follows: low birth weight (LBW); a birth weight less than 2500 g, normal birth weight; a birth weight more than 2500 g and less than 3900, low height; a height less than 47 cm, normal height; a height more than 47 cm and less than 55 cm, low head circumference; a head circumference less than 33 cm, normal head circumference; a head circumference more than 33 cm and less than 37 cm [21].

We also obtained anthropometric data (height and weight), demographic data (occupation and education), and clinical data (delivery status, IUGR and history of preterm birth, abortion, delivery status, and stillbirth) of the pregnant women. To assess participants' information regarding age and gender, marital status, and educational level, we used a standard self-reported questionnaire. Subjects' weight was measured using a balanced digital scale to the nearest 100 g, in light clothing and barefoot. Height was measured with a tape measure while the subjects were in a standing position. BMI, defined as weight in kilograms divided by height in meters squared, was calculated. General Practice Physical Activity Questionnaire (GPPAQ) was used to assess the physical activity of participants [22]. According to GPPAQ, participants were categorized into 4 levels of physical activity based on hours/week.

### 2.3. Statistical Analysis

As was mentioned, principal component analysis was used to identify major dietary patterns based on the 33 food groups. Three factors were considered major dietary patterns.

Factor scores of dietary patterns were calculated by summing intakes of foods weighed by their factor loading for each participant. Participants were categorized by quintiles of dietary pattern scores.

Continuous variables were evaluated across quintile categories of dietary pattern scores by one-way analysis of variance. Chi-square test was used to examine significant differences in the distribution of study participants in terms of categorical variables across different quintile categories of dietary pattern scores. To investigate the association between dietary patterns and neonatal anthropometric measurements, we used multivariable logistic regression models controlled for energy intake, age, and BMI. Further adjustments were made for physical activity and social-economic levels in model 2, and additionally adjustment for delivery status, IUGR, preterm delivery, history of abortion, and stillbirth were made in model 3. All statistical tests were two-sided, and P < 0.05 was considered as statistically significant. SPSS 16.0 (SPSS, Inc., Chicago, IL, USA) was used for all statistical analyses.

## 3. Results

### 3.1. Identified Major Dietary Patterns

Three major dietary patterns were identified according to the results obtained from the factor loading matrix (Table 2): (i) “western dietary pattern” which was greatly loaded by processed meats, fruit, fruit juice, citrus, nuts, fish, desserts and sweets, sugar, saturated fat, sweat fruit, potato, legumes, coffee, egg, pizza, high fat dairy, whole grain, and soft drink; (ii) “traditional dietary pattern” which was high in refined grains, colored vegetables, olive, sugar, salt, spices, unsaturated fat, garlic, onion, and tea; (iii) “healthy dietary pattern” which was high in green vegetables, leafy vegetable, colored vegetables, fruit, low-fat dairy, poultry, bulky vegetables, red meat, citrus, nuts, fish, evil, marinades, sweat fruit, egg, and unsaturated fat.

### 3.2. General Characteristics and Dietary Intakes of Study Participants

According to inclusion and exclusion criteria, 812 subjects with mean (SD) maternal age of 29.4 (4.85) remained for the current analysis. Energy and nutrients intake of the study participants across different categories of healthy, western, and traditional dietary patterns were reported in Tables 3, 4, and 5, respectively. In all dietary patterns, the most of energy and nutrients intake were different in all levels of dietary patterns adherence.

The overall characteristics of the study population across different categories of dietary patterns are presented in Table 6. Women in the highest quartile of healthy dietary pattern were more likely to be employed and graduated and have employed husband, history of IUGR, and less history of early delivery compared with those in the lowest quartile. In addition, regarding the distribution of the women across categories of western dietary pattern, it was found that those with the highest adherence had significantly higher social-economic level, employed husband, history of cesarean, and less IUGR and stillbirth history. Furthermore, women with highest quartile of traditional dietary pattern had significantly higher social-economic level, education, and husband's education compared with those in the lowest quartile.

Data in Table 7 represent the crude and adjusted odds ratio (OR) and 95% confidence interval (CI) for ORs from the multivariate analysis, where neonatal anthropometric measurements are the dependent variables and major maternal dietary patterns the independent variables. In all fitted models, the low level of each dietary pattern (quartile 1) was defined as the reference category. The comparison of neonatal height in different categories of maternal dietary patterns showed maternal dietary patterns were not related to neonatal height in all crude and adjusted models. Women in the highest quartile of adherence to traditional dietary pattern were 20% less likely to have short height infant compared with those in the lowest quartile [OR 0.80, 95% (CI) (0.34-1.85), P < 0.93] in the final adjusted model. Also, there were positive nonsignificant relationships between adherence to western [OR 1.31, 95% (CI) (0.68-2.51), P = 0.28] and healthy [OR 1.02, 95% (CI) (0.39-2.63), P= 0.40] dietary pattern of mothers with having short height infant in the final adjusted model.

Finding about neonatal weight showed that those in the top quartile of adherence to western dietary pattern had marginally significant chance of having low weight infant compared with those in the bottom quartile in the crude model [OR 1.84, 95% (CI) (0.94-3.60), P = 0.05]. The same results were obtained after adjusting for potential confounding variables in model 1 [OR 2.05, 95% (CI) (1.01-4.15), P = 0.05] and model 2 [OR 2.04, 95% (CI) (0.97-4.32), P = 0.06]. After adjusting for obstetrical factors (model 3), there was a positive significant relationship between adherence to western dietary pattern [OR 5.51, 95% (CI) (1.82-16.66), P = 0.001] with having low birth weight infant.

In addition, lower marginal significant chance of having low weight infant was found among women who were in the top adherence of traditional dietary pattern [OR 0.76, 95% (CI) (0.32-1.77), P = 0.05] in the crude model. Although this association became significant even after taking potential confounders into account in model 1 [OR 0.80, 95% (CI) (0.34-1.93), P < 0.01], such association was not seen in models 2 and 3. However, maternal adherence to healthy dietary pattern was not related to birth weight of infants in all crude and adjusted models.

We found no significant association either between maternal western dietary pattern and head circumference of infant or between healthy or traditional dietary pattern and mentioned measure. In final adjusted model, women with a higher adherence to western dietary pattern had 4.88 times larger odds for having an infant with low birth head circumference than those with a lower adherence level (OR: 4.88; 95% CI: 0.79-30.19 P = 0.35). Same result was obtained about healthy dietary pattern [OR 3.70, 95% (CI) (0.72-17.86), P = 0.52].

Furthermore, lower risk of low birth head circumference was nonsignificantly associated with a traditional dietary pattern [OR 0.58, 95% (CI) (0.13-2.51), P = 0.41].

## 4. Discussion

Current study on 812 subjects is one of the largest studies evaluating the association between major maternal dietary patterns during the first-trimester period of pregnancy and neonatal anthropometric measurements. This study demonstrated a significant association between high adherences to western dietary pattern and chance of having low birth weight infant.

However, such associations were not seen with healthy and traditional dietary pattern. Earlier studies have mostly examined the effects of particular nutrients or foods during gestational period on neonatal anthropometric measurements [13] and few data are available linking habitual diet to neonatal anthropometric measurements.

Western dietary pattern as an unhealthy food choice is unable to fulfill the increased key micronutrients requirements for normal fetal growth during pregnancy and results in such adverse effects on the fetus [23]. Earlier studies have also shown that high-quality diet assessed by using the Alternate Healthy Eating Index (AHEI) in the first trimester of pregnancy is positively associated with birth size including birth weight and birth length [15]. Other publication has also reported a protective effect of adherence to Mediterranean diet during pregnancy and risk of delivering a fetal growth–restricted infant for weight in Atlantic area [16].

In a population-based prospective study, it has been shown that women in the “wheat products” pattern had higher odds of having infants with the low birth weight and head circumference compared with women in the “rice, fish, and vegetables” pattern [24]. Also, some maternal dietary patterns characterized by high intakes of vegetable, fruit, white rice [25], Vitamin C, milk, and fat [26] are associated with larger birth size of infant. In contrast maternal intakes of carbohydrate were negatively associated with infant length and abdominal circumference [26].

Present study showed that the women with prior problematic pregnancies or higher socioeconomic status or education level were more prone to have a healthy dietary pattern. These results were in line with Northstone et al. study which showed that “health conscious” dietary patterns were negatively associated with decreasing educational level, age, and socioeconomic status [21]. Also, dietary surveys during pregnancy have suggested the poor diet quality and quantity of low socioeconomic groups [27]. It can be concluded that women with low socioeconomic status need specific dietary intervention programs due to increase in nutritional demands during pregnancy.

As was mentioned, we did not see any associations of healthy and traditional dietary patterns with consumption of red meats in the healthy dietary pattern and refined grain, salt, and sugar in the traditional dietary pattern. Mentioned foods as an unhealthy food might have been given an adverse score in the healthy and traditional dietary patterns in the current study. This might be a reason why we failed to report a significant positive association between high adherence to healthy dietary pattern and neonatal anthropometric measurements.

It should be mentioned that refined grains are major source of carbohydrate intake in Iranian diet, which has been shown to be associated with higher risk of some chronic disorders. Iranian usual diet also contains high amounts of salt, which means that salt intake among them is almost twofold the recommended levels. In addition, fruits, vegetables, and low-fat dairy intake among them is low; however, legumes intake is good in the mentioned population [28]. Therefore, understanding the differences between healthy, western, and traditional dietary patterns and usual Iranian dietary pattern might help interpreting the importance of the findings we reached.

Also, postpregnancy dietary habits and choices may be different compared with prepregnancy. The majority of pregnant women are encouraged to healthier dietary changes. We cannot overlook that the long time adherence to a specific diet affects health outcomes.

Another point that needs to be considered is the measurements error in nutritional assessment; this may increase the likelihood of misclassification, contributing to the discrepancy among our results.

Detailed information about food preparation and brands is also lacking in this instrument.

Another potential drawback should be also considered. Lack of funding to obtain fetal weights estimated via serial ultrasound is an issue in the analyses. It has been suggested that intrauterine growth patterns improved prediction of childhood anthropometry, above and beyond birth weight alone. Moreover, up to 70% of small-for-gestational-age (SGA) infants are constitutionally small (“small but healthy”), which make SGA birth a poor surrogate for intrauterine growth restriction [29]. In this regard, intrauterine growth should be identified on the basis of measures of fetal growth patterns, rather than birth weight [30].

In addition, residual confounding due to genetic backgrounds could not be excluded, although we controlled some important potential confounding factors. Therefore, these potential limitations should be considered in the interpretation of our results. However, the main strengths of the current study include its multidisciplinary scope including epidemiology, statistics, pediatrics, maternity, and nutrition. Another positive point is that few studies have collected data on maternal dietary patterns and neonatal anthropometric measurements.

In summary, evaluations carried out in the present study indicate that during early pregnancy mothers of low birth weight infant had a different dietary pattern and more adhered to the western dietary pattern compared to mothers of normal weight birth infant. Therefore, intrauterine growth conditions might be improved through the adoption of certain dietary patterns during pregnancy. Larger studies are needed to determine whether maternal dietary patterns lead to significant modifications in neonatal anthropometric measurements.

## Figures and Tables

**Table 1:** Food grouping used in the dietary patterns in early pregnancy.

**Food groups **	**Food items **
Bulky vegetables	Eggplant- Cabbage- Turnip- Mushroom- Squash- Stewed Pumpkin
Leafy vegetable	Raw Vegetables- Cooked Vegetables- Celery- Spinach- Lettuce
Colored vegetables	Tomatoes- Carrot- Pepper- Paste- Ketchup
Green vegetables	Green Peas- Green Beans - Cucumber
Garlic-onion	Garlic-Onion
Fruit	Cantaloupe-Watermelon- Pear-Apricot-Cherry Sweat-Cherry Suier Apple-Peach- Green Tomatoes- Pomegranate- Plum- Bananas
Sweat-fruit	Melon- Persimmons- Date- Fig- Grapes- Raisins- Berries- Tab and Sheet
Citrus	Kiwi-Orange-Tangerine-Lemon and Sour Lemon-Strawberry
Olive	Olive-Olive Oils
Nuts	Peanuts-Almonds-Pistachios-Hazelnuts-Roasted Seeds-Walnuts
Legumes	Beans-Peas-Lima Beans-Broad Beans-Lentils-Soy
Saturated fat	Cream-Head-Butter-Animal Oil-Mayonnaise-Solid Oil
Unsaturated fat	Liquid Oil - Olive Oil
Low fat dairy	Yogurt-Whey-Cheese-Dough- Low-Fat Milk
High fat dairy	Full Fat Milk- Chocolate Milk- Ice Cream
Whole grain	Dark Breads (Iranian Bread)-Barley Bread -Wheat Germ-Bulgur- Barley
Refined grains	White Bread (Lavash, Baguettes)-Rice-Toasted Bread-Sweet Bread White Flour –Biscuits-Corn- Macaroni- Noodle
Potato	Fries -Potato
Red meat	All Kinds of Meat- Mince Meat
Fish	Fish-Tuna
Poultry	Chicken with Or without Skin
Eggs	Eggs
Fruit juice	Fruit Juice- Lemon Juice- Compote
Soft drink	Soft Drinks
Sweets and dessert	Sweets- Gaz- Sohan- Chocolate- Halva
Marinades	Pickles- Salty
Sugar	Sugar - Candies - Sugar Cube - Honey- Jam
Salt	Salt
Spices	Spices
Pizza	Pizza
Coffee	Coffee
Tea	Tea
Processed meat	Sausages

**Table 2:** Factor loading matrix for major dietary patterns.

Foods	Dietary patterns		
	Healthy	Western	Traditional
Green vegetables	0.738	-	-
Leafy vegetables	0.708	-	-
Colored vegetables	0.652	-	0.207
Fruit	0.598	0.340	-
Dairy low fat	0.591	-	-
Poultry	0.565	-	-
Bulky vegetables	0.555	-	-
Red meat	0.438	-	-
Citrus	0.428	0.348	-
Nuts	0.396	0.395	-
Fish	0.320	0.317	-
Olive	0.301	-	0.246
Marinades	0.241	-	-
Fruit Juice	-	0.650	-
Sweets and dessert	-	0.614	-
Sugar	-	0.612	0.271
Saturated fat	-	0.558	-
Sweat fruit	0.363	0.496	-
Potato	-	0.449	-
Legumes	-	0.447	-
Coffee		0.425	-
Egg	0.339	0.380	-
Pizza	-	0.349	-
High Fat Dairy	-	0.334	-
Soft Drink	-	0.328	-
Whole Grain	-	0.287	-
Processed Meat		0.245	
Salt			0.768
Spices			0.693
Unsaturated fat	0.276		0.600
Garlic onion			0.523
Tea			0.485
Refined grain		−0.214	0.286

**Table 3:** Energy and nutrients intake of the study participants across different categories of healthy dietary pattern score.

	Healthy dietary pattern score				P value^*∗*^
	Q1 (<−0.5)	Q2 (−0.5, −0.19)	Q3 (−0.19, 0.24)	Q4 (>0.24)	
Energy (kcal/day)	1630.43±550.53^1^	1897.63±508.02	2076.23±530.73	2474.13±664.41	<0.001
Carbohydrates (g/day)	209.87±80.12	256.11±70.71	281.67±69.98	338.93±101.65	<0.001
Fat (g/day)	57.29±28.63	68.73±25	74.13±27.04	92.57±45.25	<0.001
Protein (g/day)	62.81 ±23.94	77.45±20.87	88.52±22.48	110.18±35.43	<0.001
Dietary Fiber (g/day)	22.85±11.18	29.49±10.20	33.79±10.60	38.61±14.27	<0.001
Cholesterol (mg/day)	177±91.66	233.67±82.46	254.34±87.20	306.44±141.80	<0.001
Total SFA (g/day)	18.66±10.35	23.75±8.96	24.36±9.57	29.95±15.93	<0.001
Total MUFA (g/day)	15.87±7.74	19.27±6.24	20.30±6.46	25.29±12.48	<0.001
Total PUFA (g/day)	11.11±6.63	13.13±5.72	14.36±5.87	15.46±7.72	<0.001
Vitamin B1 (mg/day)	1.08±0.41	1.28±0.32	1.48±0.39	1.99±0.74	<0.001
Vitamin B2 (mg/day)	1.56±0.74	2.07±0.54	2.36±0.58	3.28±1.60	<0.001
Vitamin B3 (mg/day)	11.84±3.88	13.78±3.63	16.24±4.11	22.53±9.46	<0.001
Vitamin B5 (mg/day)	4.28±1.65	5.57±1.36	8.65±3.76	6.19±2.76	<0.001
Vitamin B6 (mg/day)	1.31±0.44	1.65±0.32	1.88±0.36	2.74±1.33	<0.001
Vitamin B9 (*μ*g/day)	395.89±191.41	453.86±143.92	521.70±159.67	642.55±247.96	<0.001
Vitamin B12 (*μ*g/day)	3.27±1.75	4.24±1.21	4.75±1.48	6.91±4.18	<0.001
Vitamin C (mg/day)	136.99±78.20	211.77±81.71	238.95±108.07	297.12±146.35	<0.001
Vitamin D (*μ*g/day)	1.74±1.53	2.59±1.55	2.90±1.52	3.89±3.23	<0.001
Vitamin E (mg/day)	7.80±4	9.35±2.94	10.11±3.14	12.95±5.59	<0.001
Vitamin K (*μ*g/day)	166.42±135.99	236.23±141.95	296.70±215.39	521.81±386.92	<0.001
Vitamin A (RE/day)	426.54±233.91	621.44±186.20	696.85±248.03	1069.31±672.59	<0.001
Beta carotene (*μ*g/day)	2794.47±1731.83	4125.69±1759.71	4840.34±2498.58	8374.68±6517.27	<0.001
Calcium (mg/day)	914.36±493.16	1198.84±344.86	1361.07±404.54	1771±732.59	<0.001
Magnesium (mg/day)	286.50±117.87	370.13±103.19	421.14±110.60	541.76±232.07	<0.001
Fe (mg/day)	9.89±3.99	11.99±3.52	13.82±3.74	17.57±7.56	<0.001
Manganese (mg/day)	3.21±1.51	3.56±1.30	3.98±1.19	4.66±1.86	<0.001
Na (mg/day)	2827.13±1420.75	2850.06±962.77	3151.11±983.21	3779.85±1664.49	<0.001
K (mg/day)	2985.29±1218.47	4005.89±1073.81	4440.82±1136.99	5641.38±2220.18	<0.001
Zinc (mg/day)	8.99±3.41	11.27±2.85	12.86±3.32	17±7.89	<0.001
Copper (mg/day)	1.15±0.52	1.47±0.49	1.68±0.49	2.05±0.81	<0.001
Selenium (*μ*g/day)	57.68±23.44	68.40±20.85	78.82±22.87	94.49±32.08	<0.001
Chromium (*μ*g/day)	0.011±0.011	0.014±0.014	0.019±0.020	0.018±0.025	<0.001

MUFA: monounsaturated fatty acid; PUFA: polyunsaturated fatty acid; SFA: saturated fatty acid.

^*∗*^Resulted from analysis of variance.

^1^Values are presented as mean± SD.

**Table 4:** Energy and nutrients intake of the study participants across different categories of western dietary pattern score.

	Western dietary pattern score				P value^*∗*^
	Q1 (<−0.84)	Q2 (−0.84, −0.17)	Q3 (−0.17, 0.74)	Q4 (>0.74)	
Energy (kcal/day)	1664.48±597.61^1^	1770.78±589.11	2137.48±530.90	2499.67±488.63	<0.001
Carbohydrates (g/day)	211.54±83.51	244.58±89.93	289.14±76.29	341.10±70.09	<0.001
Fat (g/day)	54.20±29.34	62.58±29.57	79.67±30.17	96.24±34.33	<0.001
Protein (g/day)	71.22±33.01	75.33±28.40	89.75±28.46	102.15±25.30	<0.001
Dietary Fiber (g/day)	22.18±9.93	26.82±10.53	34.48±11.69	41.27±10.96	<0.001
Cholesterol (mg/day)	184.85±106.71	206.71±104.05	261.66±102.22	316.83±89.82	<0.001
Total SFA (g/day)	18.06±10.47	20.06±9.09	26.34±11.20	32.25±12.43	<0.001
Total MUFA (g/day)	15.67±9.26	17.06±6.58	22.08±8.35	25.91±8.76	<0.001
Total PUFA (g/day)	9.47±5.11	10.98±4.66	15.10±6.25	18.50±6.26	<0.001
Vitamin B1 (mg/day)	1.30±0.57	1.39±0.61	1.51±0.60	1.62±0.54	<0.001
Vitamin B2 (mg/day)	1.98±1.14	2.10±1.14	2.46±1.07	2.73±1.11	<0.001
Vitamin B3 (mg/day)	14.40±5.92	15.64±7.49	16.94±8.19	17.41±6	<0.001
Vitamin B5 (mg/day)	5.37±2.64	5.80±2.75	6.50±2.72	7.11±2.64	<0.001
Vitamin B6 (mg/day)	1.68±0.86	1.78±0.91	2±0.91	2.12±0.88	<0.001
Vitamin B9 (*μ*g/day)	442.48±224.57	463.44±199.86	527.60±193.20	580.48±196.49	<0.001
Vitamin B12 (*μ*g/day)	4.45±3.13	4.39±2.57	5.02±2.94	5.31±2.37	0.001
Vitamin C (mg/day)	149.67±100.08	197.06±124.74	243.65±104	294.45±105.82	<0.001
Vitamin D (*μ*g/day)	2.23±2.64	2.33±1.91	3.07±2.27	3.49±1.71	<0.001
Vitamin E (mg/day)	8.56±4.72	9.40±4.67	10.72±4.13	11.53±3.66	<0.001
Vitamin K (*μ*g/day)	293.18±269.79	294.98±266.17	323.85±316.01	309.14±248.97	<0.001
Vitamin A (RE/day)	564.08±421.46	611.98±372.25	779.70±501.18	858.37±443.16	<0.001
Beta carotene (*μ*g/day)	4313.24±3918.66	4561.48±3839.90	5614.30±4889.62	5646.16±4057.77	0.001
Calcium (mg/day)	1178.93±719.68	1215.76±611.39	1354.41±528.62	1496.17±462.83	<0.001
Magnesium (mg/day)	309.02±138.20	355.81±137.75	442.82±187.63	511.89±164.72	<0.001
Fe (mg/day)	10.62±5.50	11.86±4.80	14.32±5.64	16.47±5.09	<0.001
Manganese (mg/day)	3.14±1.49	3.54±1.58	4.12±1.43	4.61±1.42	<0.001
Na (mg/day)	2745.34±1430.81	3048.21±1476.99	3298.15±1212.62	3509.50±1114.85	<0.001
K (mg/day)	3250.01±1554.92	3729.22±1369.57	4709.18±1700.96	5374.23±1588.77	0.6
Zinc (mg/day)	10.34±4.52	11.26±5.20	13.43±6.23	15.10±5.21	<0.001
Copper (mg/day)	1.19±0.56	1.39±0.58	1.72±0.65	2.05±0.58	<0.001
Selenium (*μ*g/day)	61.89±27.44	67.61±27.55	79.18±27.72	90.72±22.46	<0.001
Chromium (*μ*g/day)	0.010±0.014	0.017±0.024	0.016±0.016	0.019±0.016	<0.001

MUFA: monounsaturated fatty acid; PUFA: polyunsaturated fatty acid; SFA: saturated fatty acid.

^*∗*^Resulted from analysis of variance.

^1^Values are presented as mean± SD.

**Table 5:** Energy and nutrients intake of the study participants across different categories of traditional dietary pattern score.

	Traditional dietary pattern score				P value^*∗*^
	Q1 (<−0.48)	Q2 (−0.48, −0.12)	Q3 (−0.12, 0.32)	Q4 (>0.32)	
Energy (kcal/day)	1801.65±650.43	2004.22±592.22	2052.10±560.61	2230.27±688.64	<0.001
Carbohydrates (g/day)	244.24±105.66	261.87±74.35	275.25±75.01	305.01±105.05	<0.001
Fat (g/day)	62.37±33.35	72.72±32.67	75.02±31.65	82.66±38.52	<0.001
Protein (g/day)	78.73±36.18	82.83±28.07	85.98±27.29	90.92±32.11	0.001
Dietary Fiber (g/day)	28.72±14.40	31.44±12.13	31.62±11	32.97±13.96	<0.001
Cholesterol (mg/day)	220.63±125.12	242.19±98.82	252.27±100.89	255.10±122.42	<0.001
Total SFA (g/day)	21.68±13.98	24.18±11.29	24.73±10.60	26.16±12.32	<0.001
Total MUFA (g/day)	17.62±9.67	19.39±8.75	20.14±6.77	23.59±10.37	<0.001
Total PUFA (g/day)	11.70±6.86	13.69±6.37	13.37±5.52	15.31±7.51	<0.001
Vitamin B1 (mg/day)	1.32±0.66	1.38±0.46	1.46±0.46	1.67±0.70	<0.001
Vitamin B2 (mg/day)	2.09±1.24	2.19±0.77	2.39±1.11	2.60±1.34	<0.001
Vitamin B3 (mg/day)	15.25±7.73	15.05±5.91	16.09±6.19	18±7.82	<0.001
Vitamin B5 (mg/day)	5.65±3.07	5.84±1.88	6.28±2.52	7.01±3.21	<0.001
Vitamin B6 (mg/day)	1.75±0.97	1.80±0.78	1.91±0.79	2.13±1.01	<0.001
Vitamin B9 (*μ*g/day)	440.44±220.02	489.13±171.98	506.64±162.56	577.79±253.04	<0.001
Vitamin B12 (*μ*g/day)	4.36±3.18	4.54±2.33	4.92±2.42	5.35±3.06	0.002
Vitamin C (mg/day)	201.36±123.75	224.14±114.97	222.56±98.81	236.77±142.37	0.03
Vitamin D (*μ*g/day)	2.57±2.53	2.77±1.72	2.94±2.18	2.83±2.38	0.4
Vitamin E (mg/day)	7.97±4.19	9.34±3.43	10.02±3.42	12.89±5.08	<0.001
Vitamin K (*μ*g/day)	300.64±303.55	278.69±229.28	316.65±280.08	325.17±285.80	<0.001
Vitamin A (RE/day)	662.33±571.37	677.87±343.67	729.23±409.11	744.70±452.74	0.2
Beta carotene (*μ*g/day)	4928.33±5105.30	4680.14±3613.57	5083.92±3937.47	5442.80±4123.45	0.3
Calcium (mg/day)	1155.88±631.37	1267.82±451.51	1370.70±588.89	1450.87±670.68	<0.001
Magnesium (mg/day)	375.19±221.24	398.22±140.64	408.83±142.54	437.29±184.32	0.005
Fe (mg/day)	12.30±6.40	13.14±5.61	13.18±4.26	14.65±6.15	<0.001
Manganese (mg/day)	3.17±1.65	3.76±1.27	3.93±1.26	4.55±1.77	<0.001
Na (mg/day)	2220.44±1055.73	3029.23±937.70	3125.09±934.55	4237.76±1529.59	
K (mg/day)	3871.97±2067.89	4193.12±1465.80	4318.04±1365.94	4678.99±1960.44	0.3
Zinc (mg/day)	11.69±6.87	12.06±4.13	12.61±4.96	13.77±6	0.001
Copper (mg/day)	1.50±0.80	1.58±0.62	1.58±0.57	1.70±0.67	0.02
Selenium (*μ*g/day)	66.58±31.15	73.55±23.94	75.70±23.97	83.56±31.82	<0.001
Chromium (*μ*g/day)	0.012±0.018	0.016±0.015	0.015±0.016	0.01±0.02	0.004

MUFA: monounsaturated fatty acid; PUFA: polyunsaturated fatty acid; SFA: saturated fatty acid.

^*∗*^Resulted from analysis of variance.

^1^Values are presented as mean± SD.

**Table 6:** General characteristics of the study participants across different categories of dietary patterns.

		**Healthy**					**Western**					**Traditional**				
		Q1	Q2	Q3	Q4	P value^*∗*^	Q1	Q2	Q3	Q4	P value^*∗*^	Q1	Q2	Q3	Q4	P value^*∗*^
BMI (kg/m^2^)	Normal	25.7%^1^	23.7%	26.5%	24.1%	0.5	25.2%	23.5%	25.2%	26.1%	0.6	24.1%	24.1%	27.2%	24.6%	0.4
	Overweight	24.2%	26.7%	23%	26.1%		24.7%	27%	24.7%	23.6%		26.1%	26.1%	22.2%	25.6%	
Mother's occupation	Housekeeper	25.3%	26.6%	25.9%	22.2%	0.01	23.9%	25.2%	25.3%	25.6%	0.4	24.5%	26.4%	25%	24.1%	0.3
	Employed	21.4%	19.8%	20.6%	38.1%		29.4%	25.4%	20.6%	24.6%		27.8%	19.8%	24.6%	27.8%	
	Teacher	28.9%	15.8%	26.3%	28.9%		26.3%	18.4%	36.8%	18.4%		23.7%	15.8%	23.7%	36.8%	
Father's occupation	Employed	17.4%	22.3%	29.3%	31%	<0.001	22.7%	23.1%	23.1%	31%	0.001	26.9%	21.9%	23.1%	28.1%	0.5
	Self-employed	27%	29.3%	22.7%	21.1%		24.5%	23.3%	26.3%	25.9%		23.6%	27%	26.3%	23.1%	
	Worker	33.9%	14.2%	25.2%	26.8%		32.3%	33.1%	23.6%	11%		26.8%	23.6%	23.6%	26%	
Mother's education	Undergraduate	25.4%	25.4%	25.1%	24%	0.9	25.7%	23.5%	27.3%	23.5%	0.4	21.3%	28.1%	27.6%	23%	0.03
	Graduate	24.8%	24.6%	24.6%	26%		24.4%	26%	23.3%	26.4%		28.2%	22.3%	23%	26.4%	
Father's education	Undergraduate	28.3%	26.7%	23.1%	21.8%	0.01	22.5%	23.8%	27.6%	26.1%	0.1	22.7%	28.5%	25.4%	23.4%	0.02
	Graduate	21.2%	22.9%	26.7%	29.2%		27.5%	26.4%	22%	24%		28.1%	20.1%	24.5%	27.3%	
Socioeconomic status	Low	28.1%	23.4%	31.2%	17.2%	0.5	32.8%	31.2%	21.9%	14.1%	0.02	12.5%	37.5%	29.7%	20.3%	<0.001
	Moderate	25.2%	27.6%	25.2%	22.1%		20.1%	21.8%	31%	27.2%		20.7%	32.3%	26.2%	20.7%	
	High	23.7%	25.6%	24%	26.6%		24.3%	24%	23.2%	28.5%		27.2%	19%	23.5%	30.3%	
Delivery	Cesarean	26.9%	24.1%	25.5%	23.4%	0.7	22.4%	22%	30.8%	24.8%	0.02	24.8%	24.8%	24.1%	26.2%	0.9
	Normal	24%	25.2%	24.8%	26%		26.5%	26.7%	21.6%	25.2%		25.2%	25%	25.4%	24.4%	
IUGR	No	25.3%	24.8%	25.8%	24.1%	0.02	23.9%	24.5%	25.9%	25.7%	0.002	24.1%	25.4%	25.2%	25.3%	0.3
	Yes	24.3%	24.3%	8.1%	43.2%		45.9%	32.4%	8.1%	13.5%		37.8%	18.9%	21.6%	21.6%	
Preterm birth	No	24.6%	25.2%	25.2%	25.1%	0.04	25.2%	24.7%	24.8%	25.3%	0.1	24.4%	24.8%	25.6%	25.2%	0.1
	Yes	52.9%	5.9%	23.5%	17.6%		17.6%	41.2%	35.3%	5.9%		41.2%	35.3%	5.9%	17.6%	
Stillbirth	No	24.8%	25.1%	25.2%	24.9%	0.16	25.2%	24.5%	25.1%	25.1%	0.01	24.8%	25%	25.2%	25%	0.3
	Yes	50%	12.5%	0%	37.5%		0%	75%	12.5%	12.5%		50%	25%	0%	25%	
Abortion	No	25.8%	25.8%	24%	24.3%	0.3	25.4%	24.6%	24.8%	25.2%	0.9	24.8%	25.1%	24.8%	25.4%	0.9
	Yes	21.6%	21.6%	29%	27.8%		23.5%	26.5%	25.9%	24.1%		25.9%	24.7%	25.9%	23.5%	
Activity	Very low	55.6%	22.2%	11.1%	11.1%	0.02	22.2%	44.4%	22.2%	11.1%	0.1	11.1%	22.2%	22.2%	44.4%	0.5
	Low	25.8%	28.9%	21.3%	24.1%		25.2%	26.2%	23.9%	24.7%		27.8%	24.3%	24.9%	23%	
	Moderate	22.5%	19.1%	31.3%	27.1%		24.8%	26%	26%	23.3%		22.5%	26.3%	25.6%	25.6%	
	High	20%	27.7%	30.8%	21.5%		23.1%	9.2%	30.8%	36.9%		16.9%	29.2%	24.6%	29.2%	

BMI: body mass index; IUGR: intrauterine growth retardation.

^*∗*^Resulted from ANOVA for quantitative variables and chi-square test for categorical data.

^1^Values are presented as percent.

**Table 7:** Multivariable adjusted odds ratios and 95% confidence intervals for neonatal anthropometric indices across quartiles of maternal dietary patterns.

	**Model **	**Healthy dietary pattern **					**Western dietary pattern **					**Traditional dietary pattern **				
		Q1	Q2	Q3	Q4	P value^*∗*^	Q1	Q2	Q3	Q4	P value^*∗*^	Q1	Q2	Q3	Q4	P value^*∗*^
Weight (kg)	Crude	1.00	1.00 (0.50-1.98)^#^	1.25 (0.65-2.40)	0.53 (0.24-1.18)	0.19	1.00	0.72 (0.32-1.60)	1.07 (0.51-2.23)	1.84 (0.94-3.60)	0.05	1.00	1.77 (0.87-3.63)	1.87 (0.92-3.80)	0.76 (0.32-1.77)	0.05
	Model 1	1.00	0.99 (0.49-2.02)	1.30 (0.66-2.56)	0.52 (0.23-1.19)	0.17	1.00	0.81 (0.35-1.86)	1.13 (0.53-2.41)	2.05 (1.01-4.15)	0.05	1.00	2.08 (0.99-4.40)	2.21 (1.06-4.61)	0.80 (0.34-1.93)	0.01
	Model 2	1.00	0.87 (0.42-1.82)	0.94 (0.45-1.94)	0.48 (0.20-1.14)	0.36	1.00	0.74 (0.30-1.80)	1.09 (0.49-2.42)	2.04 (0.97-4.32)	0.06	1.00	1.26 (0.60-2.66)	1.45 (0.70-3.30)	0.60 (0.24-1.45)	0.17
	Model 3	1.00	1.05 (0.45-2.43)	1.25 (0.53-2.91)	0.59 (0.19-1.79)	0.48	1.00	0.84 (0.29-2.40)	1.89 (0.65-5.52)	5.51 (1.82-16.6)	0.001	1.00	1.14 (0.49-2.67)	1.36 (0.58-3.19)	0.60 (0.32-1.63)	0.35
Height (cm)	Crude	1.00	0.74 (0.41-1.32)^1^	0.59 (0.32-1.09)	0.77 (0.43-1.37)	0.40	1.00	1.00 (0.53-1.87)	0.95 (0.50-1.78)	1.48 (0.82-2.66)	0.37	1.00	1.11 (0.59-2.07)	1.39 (0.76-2.53)	1.16 (0.62-2.16)	0.70
	Model 1	1.00	0.74 (0.40-1.36)	0.69 (0.36-1.30)	0.77 (0.42-1.40)	0.60	1.00	0.94 (0.49-1.81)	0.97 (0.50-1.87)	1.54 (0.83-2.83)	0.30	1.00	1.13 (0.59-2.16)	0.66 (0.88-3.12)	1.24 (0.65-2.37)	0.40
	Model 2	1.00	0.71 (0.38-1.34)	0.59 (0.29-1.17)	0.76 (0.40-1.45)	0.40	1.00	0.87 (0.43-1.76)	0.92 (0.46-1.84)	1.31 (0.68-2.51)	0.60	1.00	1.06 (0.52-2.15)	1.65 (0.83-3.30)	1.19 (0.59-2.43)	0.40
	Model 3	1.00	0.89 (0.40-1.99)	0.79 (0.33-1.88)	1.02 (0.39-2.63)	0.92	1.00	1.31 (0.53-3.23)	1.22 (0.47-3.20)	2.29 (0.86-6.13)	0.28	1.00	0.78 (0.34-1.78)	0.92 (0.41-2.07)	0.80 (0.34-1.85)	0.93
HC (cm)	Crude	1.00	1.00 (0.32-3.15)	1.88 (0.68-5.18)	1.88 (0.68-5.18)	0.40	1.00	1.83 (0.60-5.58)	1.62 (0.52-5.05)	2.49 (0.86-7.20)	0.40	1.00	1.15 (0.41-3.23)	2.07 (0.82-5.25)	0.7 (0.22-2.26)	0.15
	Model 1	1.00	0.94 (0.28-3.08)	1.98 (0.69-5.70)	1.79 (0.62-5.14)	0.38	1.00	1.91 (0.59-6.11)	1.98 (0.62-6.40)	2.56 (0.84-7.77)	0.43	1.00	1.05 (0.36-3.02)	1.95 (0.74-5.13)	0.66 (0.20-2.19)	0.19
	Model 2	1.00	0.66 (0.17-2.36)	1.55 (0.52-4.65)	1.57 (0.53-4.66)	0.44	1.00	2.01 (0.54-7.53)	2.51 (0.70-9.05)	3.18 (0.94-10.79)	0.30	1.00	0.9 (0.28-2.87)	1.96 (0.7-5.52)	0.51 (0.15-2.03)	0.13
	Model 3	1.00	1.63 (0.35-7.54)	3.35 (0.75-14.88)	3.70 (0.72-18.86)	0.52	1.00	1.93 (0.34-10.80)	2.92 (0.49-17.11)	4.88 (0.79-30.19)	0.35	1.00	0.64 (0.16-2.60)	1.48 (0.44-4.93)	0.58 (0.13-2.51)	0.41

HC: head circumference. ^*∗*^Using Mantel–Haenszel extension *χ*2 test. ^#^Odds ratios (95% confidence intervals).

**Model 1:** adjusted for energy intake, age, and BMI.

**Model 2:** additionally adjusted for physical activity and social-economic levels.

**Model 3:** additionally adjusted for delivery status, preterm delivery, IUGR, history of abortion, and stillbirth.

## References

[1] Zhang Y.-Q., Li H. (2015). Changes in weight, length, head circumference, and ponderal index at birth of healthy term newborns in nine cities in China during the period of rapid social development 1985-2005. *Economics & Human Biology*.

[2] Iheozor-Ejiofor Z., Middleton P., Esposito M., Glenny A.-M. (2017). Treating periodontal disease for preventing adverse birth outcomes in pregnant women. *Cochrane Database of Systematic Reviews*.

[3] Long H., Yi J.-M., Hu P.-L. (2012). Benefits of Iron supplementation for low birth weight infants: a systematic review. *BMC Pediatrics*.

[4] Sicard M., Nusinovici S., Hanf M. (2017). Fetal and postnatal head circumference growth: Synergetic factors for neurodevelopmental outcome at 2 years of age for preterm infants. *Neonatology*.

[5] Kuhle S., Maguire B., Ata N., MacInnis N., Dodds L. (2017). Birth weight for gestational age, anthropometric measures, and cardiovascular disease markers in children. *The Journal of Pediatrics*.

[6] Visentin S., Grumolato F., Nardelli G. B., Di Camillo B., Grisan E., Cosmi E. (2014). Early origins of adult disease: low birth weight and vascular remodeling. *Atherosclerosis*.

[7] Pham T. L., He J., Kakazu A. H., Jun B., Bazan N. G., Bazan H. E. P. (2017). Defining a mechanistic link between pigment epithelium–derived factor, docosahexaenoic acid, and corneal nerve regeneration. *The Journal of Biological Chemistry*.

[8] Hirano D., Ishikura K., Uemura O. (2016). Association between low birth weight and childhood-onset chronic kidney disease in Japan: A combined analysis of a nationwide survey for paediatric chronic kidney disease and the National Vital Statistics Report. *Nephrology Dialysis Transplantation *.

[9] Chen P., Chu A., Liao W. (2017). Prenatal growth patterns and birthweight are associated with differential DNA methylation and gene expression of cardiometabolic risk genes in human placentas: a discovery-based Approach. *Reproductive Sciences*.

[10] Sferruzzi-Perri A. N., Sandovici I., Constancia M., Fowden A. L. (2017). Placental phenotype and the insulin-like growth factors: resource allocation to fetal growth. *The Journal of Physiology*.

[11] Wrottesley S. V., Lamper C., Pisa P. T. (2016). Review of the importance of nutrition during the first 1000 days: maternal nutritional status and its associations with fetal growth and birth, neonatal and infant outcomes among African women. *Journal of Developmental Origins of Health and Disease*.

[12] Seo W. H., Choi B. M., Lee H., Yoo Y., Choung J. T., Han Y. (2017). Effect of the prenatal maternal environments and diets on cord blood interleukin-4 and interferon -gamma: a pilot study. *Asian Pacific Journal of Allergy and Immunology*.

[13] Murphy M. M., Stettler N., Smith K. M., Reiss R. (2014). Associations of consumption of fruits and vegetables during pregnancy with infant birth weight or small for gestational age births: A systematic review of the literature. *International Journal of Women's Health*.

[14] Naghavi S., Mahdavi S. B., Moradi B., Tajadini M. H., Kelishadi R. (2017). Association of dietary patterns during pregnancy and cord blood nitric oxide level with birth weight of newborns. *International Journal of Pediatrics*.

[15] Rodríguez-Bernal C. L., Rebagliato M., Iñiguez C. (2010). Diet quality in early pregnancy and its effects on fetal growth outcomes: The infancia y medio ambiente (childhood and environment) mother and child cohort study in Spain. *American Journal of Clinical Nutrition*.

[16] Chatzi L., Mendez M., Garcia R. (2012). Mediterranean diet adherence during pregnancy and fetal growth: INMA (Spain) and RHEA (Greece) mother-child cohort studies. *British Journal of Nutrition*.

[17] Hu F. B. (2002). Dietary pattern analysis: a new direction in nutritional epidemiology. *Current Opinion in Lipidology*.

[18] Kant A. K., Graubard B. I., Schatzkin A. (2004). Dietary patterns predict mortality in a national cohort: the National Health Interview Surveys, 1987 and 1992. *Journal of Nutrition*.

[19] Rafieifar S., Pouraram H., Djazayery A. (2016). Strategies and opportunities ahead to reduce salt intake. *Archives of Iranian Medicine*.

[20] Hashemi R., Motlagh A. D., Heshmat R. (2015). Diet and its relationship to sarcopenia in community dwelling iranian elderly: A cross sectional study. *Nutrition Journal *.

[21] Northstone K., Emmett P., Rogers I. (2008). Dietary patterns in pregnancy and associations with socio-demographic and lifestyle factors. *European Journal of Clinical Nutrition*.

[22] For Nursing NCC, The General Practice Physical Activity Questionnaire (GPPAQ), 2008

[23] Knudsen V. K., Orozova-Bekkevold I. M., Mikkelsen T. B., Wolff S., Olsen S. F. (2008). Major dietary patterns in pregnancy and fetal growth. *European Journal of Clinical Nutrition*.

[24] Okubo H., Miyake Y., Sasaki S. (2012). Maternal dietary patterns in pregnancy and fetal growth in Japan: the Osaka Maternal and Child Health Study. *British Journal of Nutrition*.

[25] Chia A.-R., De Seymour J. V., Colega M. (2016). A vegetable, fruit, and white rice dietary pattern during pregnancy is associated with a lower risk of preterm birth and larger birth size in a multiethnic Asian cohort: The Growing Up in Singapore Towards healthy Outcomes (GUSTO) cohort study. *American Journal of Clinical Nutrition*.

[26] Hjertholm K. G., Iversen P. O., Holmboe-Ottesen G. (2018). Maternal dietary intake during pregnancy and its association to birth size in rural Malawi: A cross-sectional study. *Maternal & Child Nutrition*.

[27] Doyle W., Crawford M. A., Laurance B. M., Drury P. (1982). Dietary survey during pregnancy in a low socio-economic group. *Human Nutrition: Applied Nutrition*.

[28] Valipour G., Esmaillzadeh A., Azadbakht L., Afshar H., Hassanzadeh A., Adibi P. (2017). Adherence to the DASH diet in relation to psychological profile of Iranian adults. *European Journal of Nutrition*.

[29] No GtG, The investigation and management of the small–for–gestational–age fetus, 2002

[30] Hutcheon J. A., Jacobsen G. W., Kramer M. S., Martinussen M., Platt R. W. (2016). Small size at birth or abnormal intrauterine growth trajectory: Which matters more for child growth?. *American Journal of Epidemiology*.

